# Are we there yet? Benchmarking low-coverage nanopore long-read sequencing for the assembling of mitochondrial genomes using the vulnerable silky shark *Carcharhinus falciformis*

**DOI:** 10.1186/s12864-022-08482-z

**Published:** 2022-04-22

**Authors:** J. Antonio Baeza, F. J. García-De León

**Affiliations:** 1 Department of Biological Sciences, 132 Long Hall, Clemson University, Clemson, SC 29634 USA; 2 Smithsonian Marine Station at Fort Pierce, 701 Seaway Drive, Fort Pierce, Florida, 34949 USA; 3 Departamento de Biología Marina, Facultad de Ciencias del Mar, Universidad Católica del Norte, Larrondo, 1281 Coquimbo, Chile; 4 Laboratorio de Genética para la Conservación, Centro de Investigaciones Biológicas del Noroeste, S.C., La Paz, Baja California Sur Mexico

**Keywords:** Long-read sequencing, Nanopore, Elasmobranch

## Abstract

**Background:**

Whole mitochondrial genomes are quickly becoming markers of choice for the exploration of within-species genealogical and among-species phylogenetic relationships. Most often, ‘primer walking’ or ‘long PCR’ strategies plus Sanger sequencing or low-pass whole genome sequencing using Illumina short reads are used for the assembling of mitochondrial chromosomes. In this study, we first confirmed that mitochondrial genomes can be sequenced from long reads using nanopore sequencing data exclusively. Next, we examined the accuracy of the long-reads assembled mitochondrial chromosomes when comparing them to a ‘gold’ standard reference mitochondrial chromosome assembled using Illumina short-reads sequencing.

**Results:**

Using a specialized bioinformatics tool, we first produced a short-reads mitochondrial genome assembly for the silky shark *C. falciformis* with an average base coverage of 9.8x. The complete mitochondrial genome of *C. falciformis* was 16,705 bp in length and 934 bp shorter than a previously assembled genome (17,639 bp in length) that used bioinformatics tools not specialized for the assembly of mitochondrial chromosomes. Next, low-pass whole genome sequencing using a MinION ONT pocket-sized platform plus customized *de-novo* and *reference-based* workflows assembled and circularized a highly accurate mitochondrial genome in the silky shark *Carcharhinus falciformis*. Indels at the flanks of homopolymer regions explained most of the dissimilarities observed between the ‘gold’ standard reference mitochondrial genome (assembled using Illumina short reads) and each of the long-reads mitochondrial genome assemblies. Although not completely accurate, mitophylogenomics and barcoding analyses (using entire mitogenomes and the D-Loop/Control Region, respectively) suggest that long-reads assembled mitochondrial genomes are reliable for identifying a sequenced individual, such as *C. falciformis*, and separating the same individual from others belonging to closely related congeneric species.

**Conclusions:**

This study confirms that mitochondrial genomes can be sequenced from long-reads nanopore sequencing data exclusively. With further development, nanopore technology can be used to quickly test in situ mislabeling in the shark fin fishing industry and thus, improve surveillance protocols, law enforcement, and the regulation of this fishery. This study will also assist with the transferring of high-throughput sequencing technology to middle- and low-income countries so that international scientists can explore population genomics in sharks using inclusive research strategies. Lastly, we recommend assembling mitochondrial genomes using specialized assemblers instead of other assemblers developed for bacterial and/or nuclear genomes.

## Background

Entire or partially complete mitochondrial genomes are quickly becoming markers of choice for examining phylogenetic relationships [1–10]. The appeal for using entire (or partially complete) mitochondrial genomes has to do with its nearly neutral fashion of molecular evolution as well as its mutation rate that is high compared to that of most nuclear markers [1, 11], (but see [12]). Furthermore, extraction, purification, and sequencing of mitochondrial DNA is straightforward. The mitochondrial genome also behaves as a single non-recombining locus because mitochondrial inheritance is maternal-only (clonal) (but see [2, 13]).

The customary approach for sequencing and assembling partial or entire mitochondrial genomes has historically relied on ‘long PCR’ or ‘primer walking’ and cloning plus Sanger sequencing [14]. Most recently, however, 2nd generation sequencing technologies (i.e., Illumina short reads) have been used to assemble complete mitochondrial genomes using low-pass (=low-coverage) whole genome sequencing (WGS) [6]. The aforementioned strategy almost invariably results in the assembly of complete and fully accurate mitochondrial genomes. Nonetheless, the main problem with the use of short reads for assembling mitochondrial genomes is that it is time demanding; from gDNA extraction to mitochondrial genome assembly, studies can take weeks, months, or even years [4–10]. Mitochondrial genome sequencing approaches that rely solely on Illumina short reads are not the optimal solution for studies that demand the speedy recovery of molecular markers, including complete mitochondrial genomes. Such studies include, among others, the in-situ detection of mislabeling in the supply chain (either legal or illegal) of biological commodities [15] and the real-time genomic surveillance of disease agents [16].

The use of 3rd generation sequencing technology (e.g., long reads from Oxford Nanopore Technologies [ONT] or Pacific Biosciences [PacBio] platforms) represents an alternative to short-read sequencing for assembling complete mitochondrial genomes. Currently, third generation sequencing technology yields molecules as long as mitochondrial genomes (i.e., ~ 10–20 kbp and up to 1–2 Mbp – 17). However, the initial sequence error rate of 3rd generation sequencing technology is high (PacBio = 11–15%; ONT = 5–15% - 20, 21) and much greater than Illumina sequencing (0.3% - 18, 19). *In-silico* read ‘polishing’ algorithms have been developed to correct for the high initial error rate of long reads (i.e., nanopore – [17] and references therein). Assembling complete and accurate mitochondrial genomes using 3rd generation sequencing exclusively should be straightforward because they are short, circular, non-repetitive, haploid genomes.

Currently, only three studies have employed nanopore long reads exclusively for the de novo assembly of complete mitochondrial genomes: in the cosmopolitan silky shark *Carcharhinus falciformis* [18], in the neotropical rodent *Melanomys caliginosus* [19], and in the Caribbean spiny lobster *Panulirus argus* [20]. Other studies have used both short- and long-reads datasets concomitantly for the ‘hybrid assembly’ of mitochondrial genomes [21, 22], (see also [23–26]). Importantly, among the studies assembling mitochondrial genomes with long reads exclusively, only the spiny lobster study benchmarked the long-reads assembled mitochondrial genome with a short-reads mitochondrial genome assembly generated from the same individual; the comparison revealed that long reads can assemble complete and highly accurate, but not perfect, mitochondrial genomes [20]. The *Carcharhinus falciformis* study did not successfully benchmark the long-reads mitochondrial assembly, but in the *M. caliginosus* study it was benchmarked using two short protein coding gene fragments [19]. Benchmarking of long-reads assemblies with full reference genomes is of paramount significance given the high initial error rate of 3rd generation sequencing technologies. This information will assist with the optimization of bioinformatics workflows for the de novo assembly of mitochondrial genomes.

In this study, we are interested in benchmarking long-reads assembled mitochondrial genomes, and confirming the utility of 3rd generation sequencing technologies for the rapid sequencing and assembling of relatively short (i.e., mitochondrial) genomes. For this purpose, we used the silky shark *Carcharhinus falciformis* as a model system, a large and highly migratory shark with a circumglobal distribution in tropical and subtropical oceanic and coastal-pelagic waters [27, 28]. *Carcharhinus falciformis* is one of the most commonly fished sharks worldwide and is targeted by both regional and international fisheries [29]. It also comprises a large portion of the bycatch in fisheries targeting tunas (*Thunnus* spp.) around the world [29–32]. Fishing pressure appears to have resulted in steady silky shark population declines therefore, the species has been classified globally as vulnerable since 2017 by the International Union for Conservation of Nature [33]. The silky shark was also added to Appendix II of the Convention on International Trade in Endangered Species the same year [33].

Despite its vulnerable status, only a few (but increasing) number of genomic resources exist for this species [18, 34–37]. The mitochondrial genome of *C. falciformis* was assembled using short-reads by Galván-Tirado et al. [34]. An unusual insertion ~ 939 bp in length was detected in this short-reads assembled mitochondrial genome after comparison to other congeneric sharks, whose mitochondrial genomes are usually ~ 16,700 bp in length. The bioinformatics pipeline used in [34] was not specifically developed for assembling mitochondrial genomes, thus, the odd insertion could be a bioinformatics artifact. Most recently, Johri et al. [18, 35] used nanopore long reads exclusively to assemble the mitochondrial genome of this species and did not find a long insertion. The length of the long-reads mitochondrial assembly was similar to that of other mitochondrial genomes in *Carcharhinus* spp. The authors claimed that the long-reads assembly was highly accurate. However, no benchmarking of this assembly was conducted and the authors provided no information about the algorithm used for final assembly curation [18, 35].

To accomplish the aims of this study, we first attempted to assemble a high-quality, gold-standard mitochondrial genome for *C. falciformis* using Illumina short reads and a specialized bioinformatics pipeline exclusively developed for the retrieval of entire mitochondrial genomes. We used the same dataset from Galván-Tirado et al. [34] to determine if the unusually long insertion observed in the first assembly by these authors was a by-product of using a bioinformatics pipeline not customized for the assembly of mitochondrial genomes. Second, we de novo assembled the mitochondrial genome of *C. falciformis* using long reads exclusively and benchmarked the accuracy of these long-reads assembled genomes by comparing them to the ‘gold’ standard mitochondrial genome assembled using short-reads (Illumina) sequencing data. To achieve this second goal, we used the same dataset from Johri et al. [18, 35] but employed different de novo and *reference-based* bioinformatics pipelines specifically developed for the rapid retrieval of mitochondrial genomes using long reads exclusively [20], (see also [38]). The sequence accuracy of the long-reads assemblies was explored with multiple metrics; completeness, identity, and coverage, as in [20]. A detailed quantitative analysis of error type in long-reads assemblies was conducted. Finally, we explored whether or not de novo and *reference-based* long-reads mitochondrial genome assemblies are useful for mitophylogenomics and barcoding research.

## Results

### Assembly of the mitochondrial genome using short reads

**Fig. 1:**
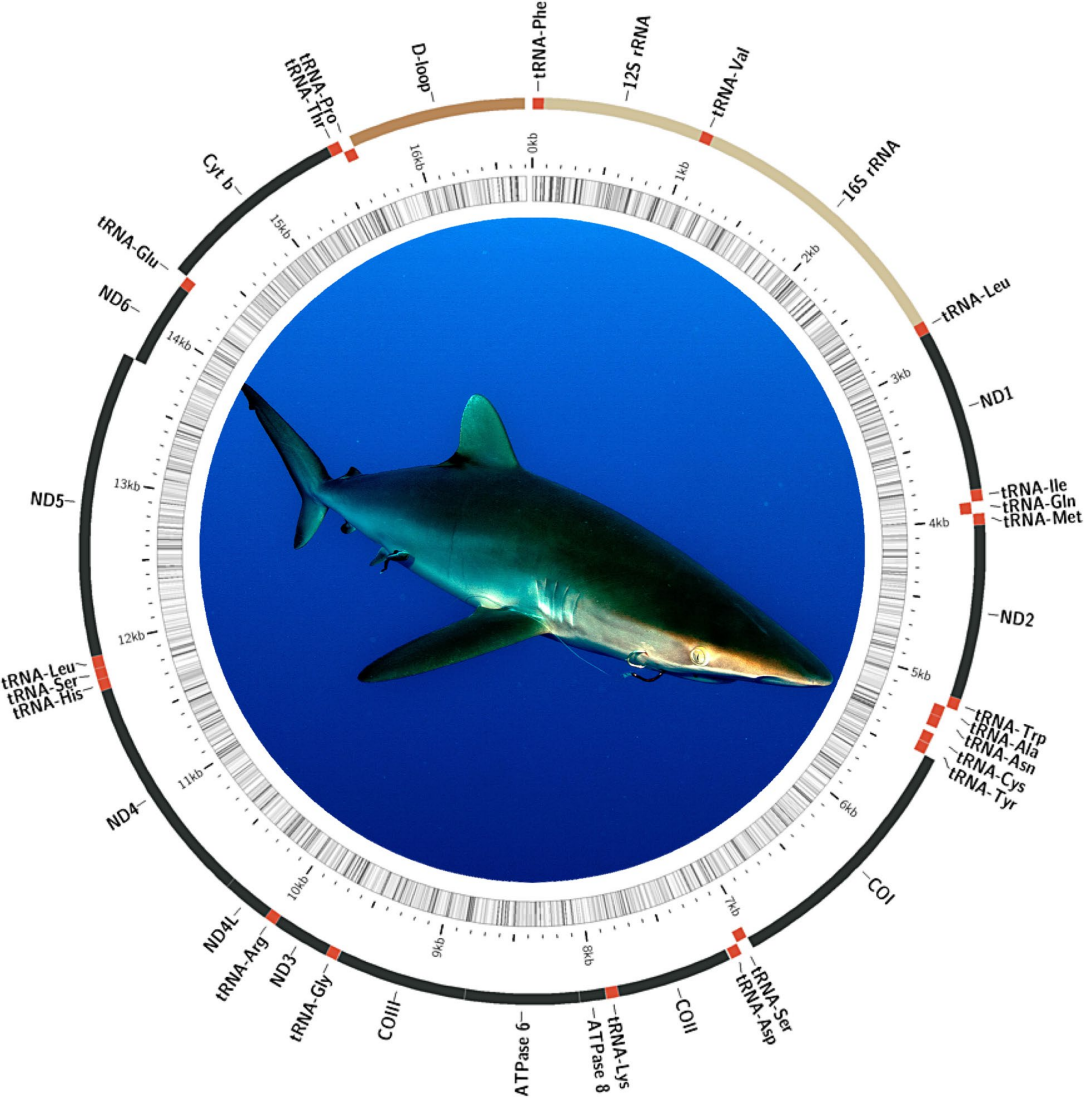
Circularized mitochondrial genome ideogram of the silky shark *Carcharhinus falciformis*. The map is annotated and depicts a single putative control region, 22 transfer RNA (tRNA) genes, 2 ribosomal RNA genes (rrnS [12S ribosomal RNA] and rrnL [16S ribosomal RNA]), and 13 protein-coding genes (PCGs). Shark photograph: Joi Ito (Attribution 2.0 Generic [CC BY 2.0])

The software GetOrganelle [39] assembled and circularized the mitochondrial genome of the silky shark *C. falciformis* with an average base coverage of 9.8x (Fig. 1). The complete mitochondrial genome of *C. falciformis* was 16,705 bp in length (OM885432) and 934 bp shorter than the previously assembled genome, 17,639 bp in length (KF801102), which used SOAP de novo [34].

Annotation in MITOS2 [40] and MitoFish [41] indicated that the mitochondrial genome of *C. falciformis* encoded 22 transfer RNA (tRNA) genes, 2 ribosomal RNA genes (rrnS [12S ribosomal RNA] and rrnL [16S ribosomal RNA]), and 3 protein-coding genes (PCGs). All but one PCG (*nad6*) and 14 tRNA genes were encoded on the H-strand (Fig. 1). The two ribosomal RNA genes were also encoded in the H-strand. The D-loop/Control Region was assumed to be a relatively long inter-genic space, 1065 bp long, in the mitochondrial genome of *C. falciformis*. Mitochondrial synteny observed in *C. falciformis* is identical to that reported before in the genus *Carcharhinus* ([34] and references therein).

### Assembly of the mitochondrial genome using long reads

The mitochondrial genome of *C. falciformis* was assembled and circularized by all used bioinformatics pipelines: Unicycler [42], Flye [43], and Rebaler [44], and with or without ‘extra’ polishing with the software Medaka (see [45]). Assembled contigs by each of the pipelines above identified as circular with the program Bandage [46] matched the mitochondrial genome of *C. falciformis* and other congeneric species available in NCBI’s GenBank after blasts against the nucleotide non-redundant database (all e-values << 1e^− 10^).

All long-reads assemblies, either de novo (i.e., Unicycler and Flye) or *reference-based* (i.e., Rebaler), before extra polishing using the software Medaka, varied in length between 16,690 bp (Flye with 1 polishing cycle) and 16,801 bp (Unicycler Normal, Bold, and Conservative). Interestingly, the long-reads mitochondrial genomes assembled with Flye were shorter than the reference genome assembled with short reads. However, the mitochondrial genomes assembled with Unicycler and Rebaler were longer than the reference genome (Table 1). Furthermore, all mitochondrial genomes extra-polished with Medaka were shorter than the same assemblies before extra-polishing using Medaka was applied to them (Table 1).

**Table 1:** Accuracy metrics for different de novo and *reference-based* mitochondrial genome assemblies using nanopore long reads exclusively in the silky shark *Carcharhinus falciformis*

Assembly Pipeline	Contigs	Length	Coverage	p-dist	Errors^b^
Flye +1p	circular	16,690	20x	0.001023172	65
Flye +1p + Medaka	circular	16,475	20x	0.000541679	70
Flye +5p	circular	16,691	20x	0.001023172	69
Flye +5p + Medaka	circular	16,475	20x	0.000541679	71
Flye +10p	circular	16,691	20x	0.001023172	69
Flye +10p + Medaka	circular	16,475	20x	0.000541679	71
Unicycler - N	circular	16,801	2.28x^a^	0.001143545	110
Unicycler - N + Medaka	circular	16,781	2.28x^a^	0.000601866	89
Unicycler - B	circular	16,801	2.28x^a^	0.001143545	110
Unicycler - B + Medaka	circular	16,781	2.28x^a^	0.000601866	89
Unicycler - C	circular	16,801	2.28x^a^	0.001143545	110
Unicycler - C + Medaka	circular	16,781	2.28x^a^	0.000601866	89
Rebaler - *P. amblyrhynchos*	circular	15,782	50.59x	0.001324105	106
Rebaler - *P*. *ambly*. + Medaka	circular	16,774	50.59x	0.000541679	81
Rebaler - *P. amboinensis*	circular	15,790	49.94x	0.000902799	95
Rebaler - *P*. *ambo.* + Medaka	circular	16,776	49.94x	0.000361119	73
Rebaler - *P. falciformis*	circular	16,789	52.52x	0.000842612	96
Rebaler - *P*. *falci.* + Medaka	circular	16,777	52.52x	0.000541679	81
Reference mtDNA	circular	16,705	9.8x	–	–

^a^Unicycler normalizes the depth of contigs to the median value

^b^Error refers to total number of errors quantified in the long-read assemblies compared to the short read assembly. Errors were classified as single, double, triple, quadruple, quintuple, sextuple, or septuple “homopolymer insertions’ or ‘homopolymer deletions’, ‘simple substitution’, ‘single insertion’, ‘short insertion (< 5 bp)’, ‘single deletion’, and ‘short deletion (< 5 pb)’

Alignment of the different long-reads assemblies to the reference genome and subsequent p-distance estimation revealed that long-reads assemblies were either identical (i.e., Unicycler Normal = Unicycler Conservative = Unicycler Bold; Unicycler Normal + Medaka = Unicycler Conserative + Medaka = Unicycler Bold + Medaka; Flye with 5 polish rounds = Flye with 10 polish rounds; Flye with 1 polish round + Medaka = Flye with 5 polish rounds + Medaka = Flye with 10 polish rounds + Medaka) or very similar to each other with p-distances that ranged between 1.203732 × 10^− 4^ and 1.6250376 × 10^− 3^ when dissimilar.

Identity, estimated as p-distance between the short-reads assembly versus a specific long-reads assembly, was also very high; all long-reads assemblies were not identical but a close match to the reference short-reads mitochondrial genome with p-distances ranging between 3.611195 × 10^− 4^ (reference compared to Rebaler using *C. amboinensis* as a reference + Medaka) and 1.3241047 × 10^− 3^ (reference compared to Rebaler using *C. amblyrhynchos* as a reference) (Table 1).

### Error estimation in long-read assembled mitochondrial genomes in the silky shark

**Fig. 2:**
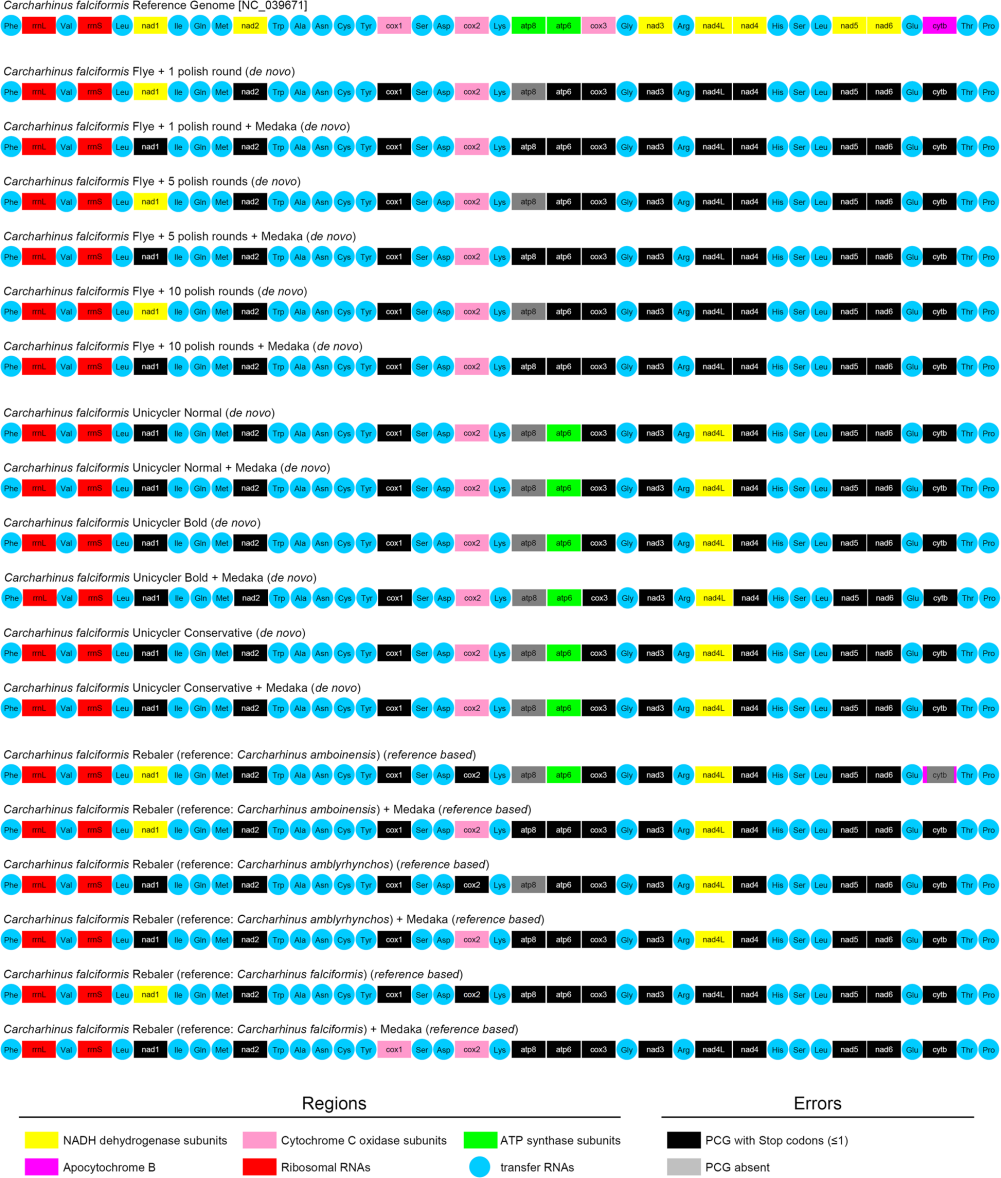
Sequence errors per de novo (Unicycler and Flye) and *reference-based* assemblers (Rebaler) without and with ‘extra polishing’ using the program Medaka for the silky shark *Carcharhinus falciformis* mitochondrial genome. Benchmarking of all long-read assemblies occurred against the Illumina short-read assembly (‘gold’ standard)

Discordance between the reference assembly and each of the long-reads assemblies was mostly due to indels at the flanks of homopolymer regions comprised of all four nucleotide types (Fig. 2). By far, the most common errors identified in all long-reads assemblies were single nucleotide homopolymer insertions (range = 18 errors in the Flye + 1 polish assembly to 60 errors in all three Unicycler assemblies polished with Medaka; aggregate number of errors in all assemblies [n_T_] = 716) followed by single nucleotide homopolymer deletions (n_T_ = 240) and double nucleotide homopolymer insertions (n_T_ = 177). Errors due to single substitutions (n_T_ = 105), single deletions (n_T_ = 69), and single insertions (n_T_ = 65), were moderately abundant. Triple, quadruple, quintuple, sextuple, and septuplet nucleotide homopolymer insertions were much less common (Fig. 2). Similarly, short oligonucleotide deletions and double homopolymer deletions were not common. We did not observe errors due to triple, quadruple, quintuple, sextuple, and septuplet nucleotide homopolymer deletions in any of the long-read assemblies (Fig. 2).

The effect of extra-polishing long-reads assemblies with the program Medaka was not homogenous across assembly pipelines. For instance, a decrease in the number of errors (mostly single nucleotide homopolymer deletions and double nucleotide homopolymer insertions) after Medaka extra-polishing was evident for mitochondrial genomes assembled with the de novo assembly pipeline Unicycler (all three strategies) and the *reference-based* assembly pipeline Rebaler (all three strategies) (Fig. 2). However, for mitochondrial genomes assembled with the de novo assembly pipeline Flye, the total number of errors increased slightly when Medaka extra-polishing was applied to them. In the Flye assemblies (all strategies), Medaka extra-polishing decreased the number of errors due to single nucleotide homopolymer deletions but disproportionally increased the number of single nucleotides homopolymer insertions, explaining the slight increase in overall assembly error observed in these Medaka extra-polished Flye assemblies. In general, extra-polishing with Medaka resulted in increased accuracy for mitochondrial genomes assembled with the pipelines Rebaler and Unicycler but not with the program Flye.

Overall, accuracy of the long-reads assemblies was similar when assessed in terms of completeness (circularization), coverage, length, identity, and sequence errors. Additionally, long-reads genome accuracy was very high (but not perfect; < 100%) when compared to the short-reads assembled mitochondrial genome herein used as a gold standard (Fig. 2; Table 1).

### Annotation of mitochondrial genomes assembled with long reads

**Fig. 3:**
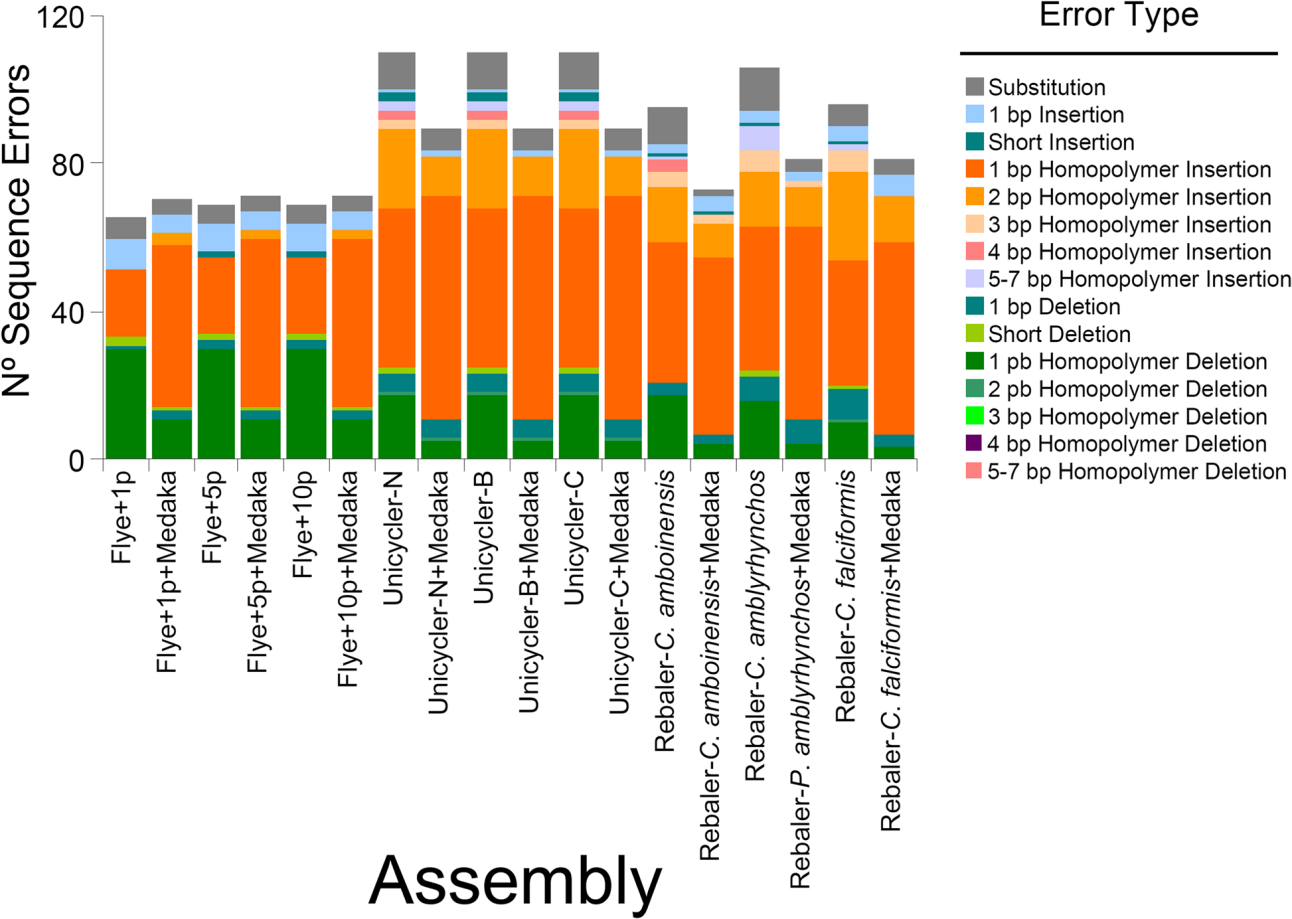
Annotation of *reference-based* (Rebaler) and de novo (Fyer and Unicycler) mitochondrial genomes assembled using long reads in the silky shark *Carcharhinus falciformis*. Assemblies depicted include those with and without ‘extra polishing’ with the program Medaka

Annotation of the de novo and *reference-based* long-reads assembled mitochondrial genomes, with or without extra-polishing with Medaka, demonstrated that synteny and gene number were either identical or very similar to that of the reference genome (Fig. 3). In all long-reads assemblies, 9 to 12 PCGs had at least one internal, but often more, stop codon that disrupted their open reading frames. In several of the long-reads assemblies (*n* = 11), the relatively short PCG *atp8* was not detected by the in-silico annotation tools. However, manual curation demonstrated that this short gene was present in all the assemblies but disrupted due to the occurrence of stop codons (Fig. 3). Overall, even though all long-reads assemblies were highly accurate, the errors contained in each long-reads assembled mitochondrial genome precluded generating a reliable annotation with MITOS2 and MitoFish (Fig. 3).

### Mitophylogenomics using long-read mitochondrial genome assemblies

**Fig. 4:**
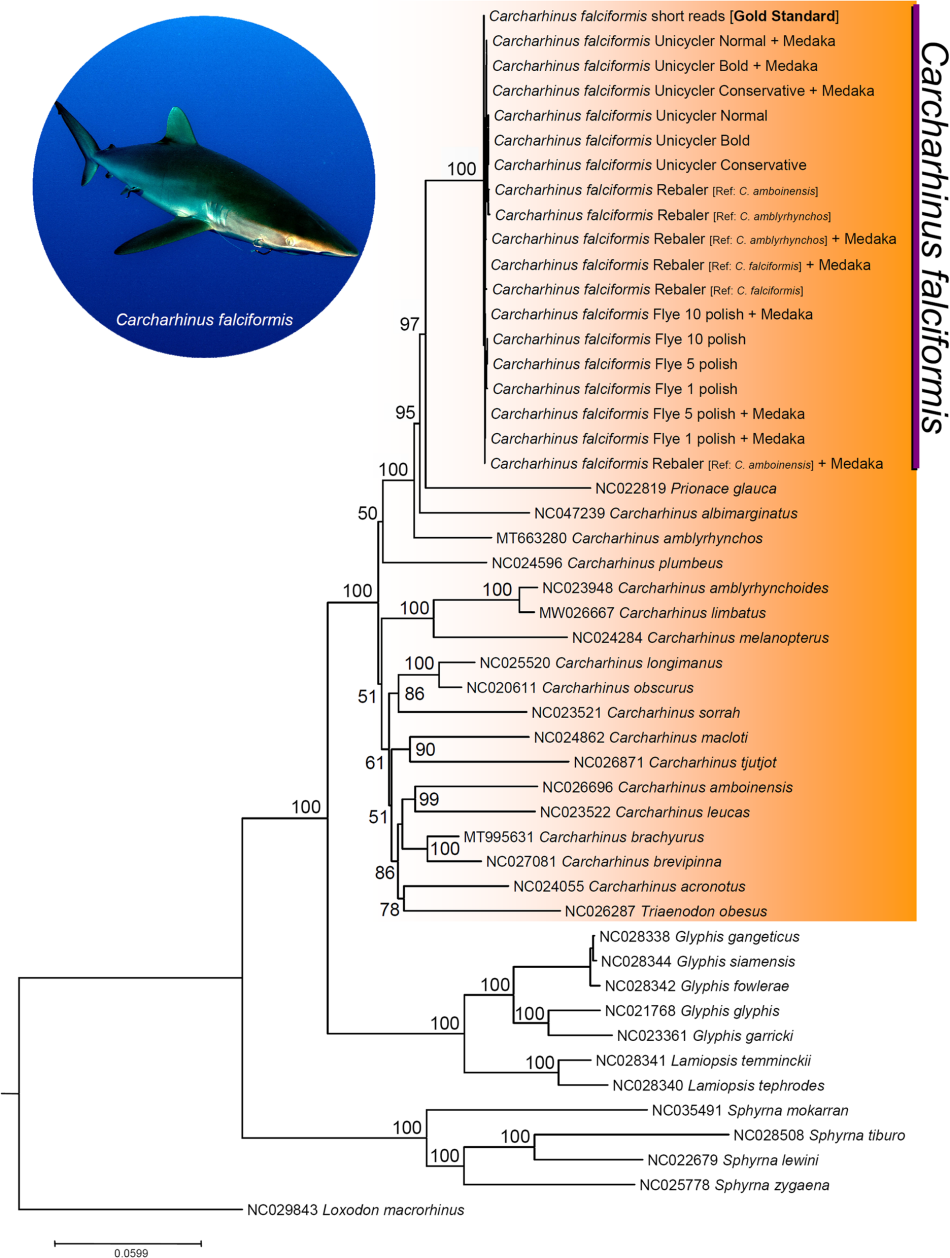
Mitophylogenomic analysis of the genus *Carcharhinus* and allies, including mitochondrial genomes of the silky shark *Carcharhinus falciformis* assembled with long reads exclusively and short reads (‘gold standard’). Nodes with bootstrap support values > 90 are marked with an orange circle. Shark photograph: Joi Ito (Attribution 2.0 Generic [CC BY 2.0])

In the phylogenetic tree resulting from the ML analysis (49 terminals, 14,245 nucleotide characters, 3396 parsimony informative sites), the short-reads assembled reference genome plus all of the long-reads assembled mitochondrial genomes (*n* = 18) clustered together into a single, fully supported monophyletic clade (bootstrap value [bv] = 100) (Fig. 4). Interestingly, the phylogenetic analysis did not confirm the monophyletic status of the genus *Carcharhinus*. The blue shark *Prionace glauca* and the whitetip reef shark *Triaenodon obesus* clustered together in the same fully supported clade containing all the representatives belonging to the genus *Carcharhinus*. Specifically, the tree placed *P. glauca* in a position sister to *C. falciformis* (short-reads reference + all long-reads assemblies) while *T. obesus* comprised a moderately supported (bv = 86) monophyletic clade with *C. amboinensis*, *C. acronotus*, *C. brachyurus*, *C. brevipinna*, and *C. leucas* (Fig. 4). Support values did not decrease towards the root of the phylogenetic tree. The above suggests that mitochondrial genomes alone will likely have enough phylogenetic information to reveal relationships at higher taxonomic levels within the family Carcharhinidae, including the genus *Carcharhinus* and other closely related genera (i.e., *Glyphis*, *Laminopsis*, *Sphyrna*).

### Barcoding using long-reads assemblies

**Fig. 5:**
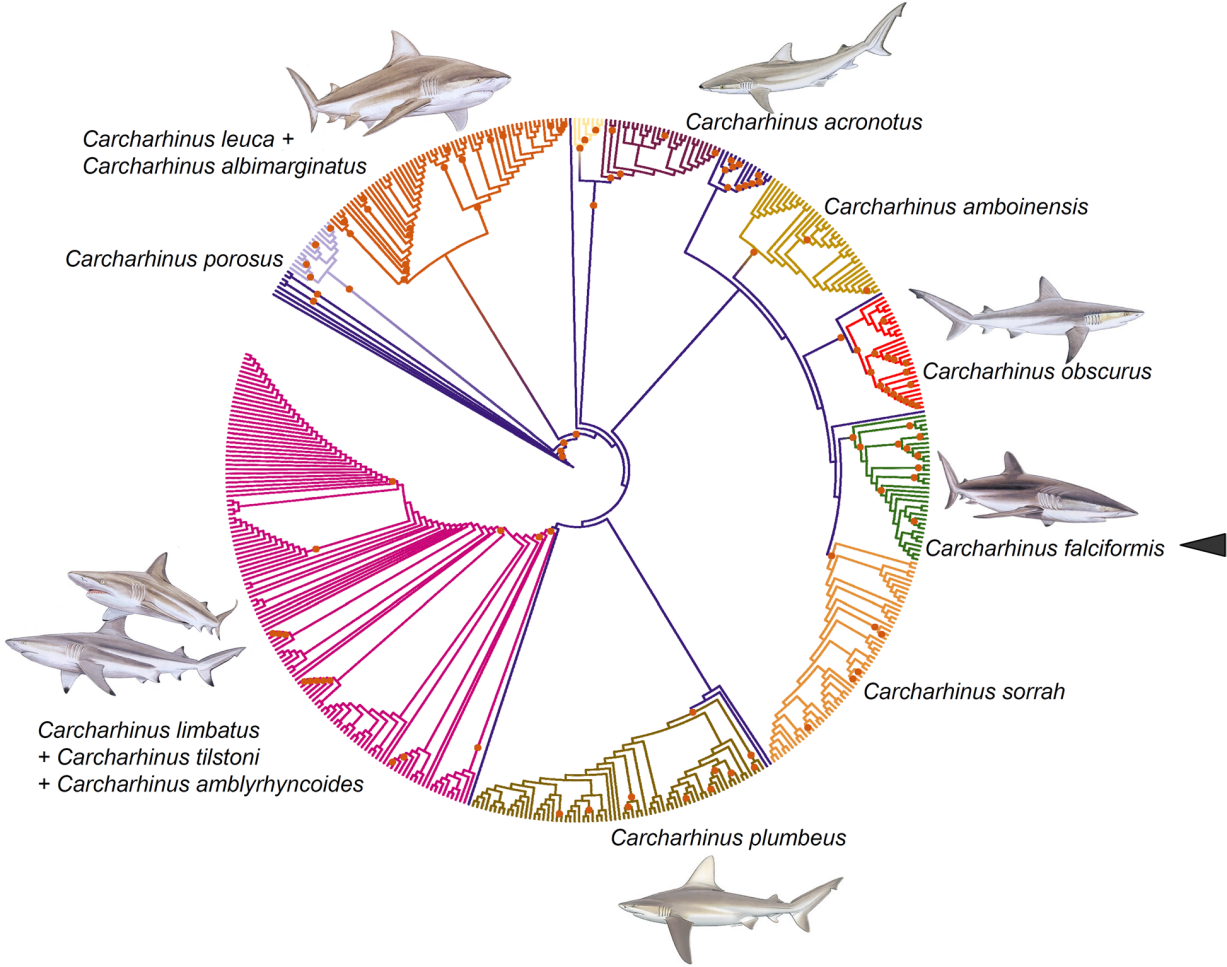
Barcoding analysis of the genus *Carcharhinus* using the D-Loop/Control Region (CR), including the CR retrieved from mitochondrial genomes of the silky shark *Carcharhinus falciformis* mitochondrial genome assembled with long reads alone and short reads (‘gold standard’) plus 447 other specimens belonging to the genus *Carcharhinus* retrieved from Genbank. Shark drawings from M. Dando (used with permission) [47]

In the barcoding analysis based on the Control Region, the aligned molecular data matrix was comprised of 1188 characters, of which 570 were parsimony informative. A total of 476 terminals belonged to sharks in the genus *Carcharhinus*, other related confamilial species (*Prionace glauca* [*n* = 1] and *Triaenodon obesus* [n = 1]), outgroup terminals from the genera *Glyphis* (*n* = 5 terminals) and *Sphyrna* (*n* = 4), plus *Galeocerdo cuvier* (n = 5) and *Loxodon macrorhynus* (n = 1) (Fig. 5). In the ML molecular phylogenetic tree (Fig. 5), the Control Region fragment retrieved from the short-reads assembled reference genome plus the totality (*n* = 18) of the long-reads assembled mitochondrial genomes and 15 other sequences belonging to *C. falciformis* retrieved from Genbank clustered together into a fully supported (bv = 100) monophyletic clade (Fig. 5). Other fully or well supported clades in the analysis included the dusky shark *C. obscurus*, the spot-tail shark *C. sorrah*, the sandbar shark *C. plumbeus*, the pig-eye or java shark *C. amboinensis*, the black-nose shark *C. acronotus*, the fine-tooth shark *C. isodon*, and the small-tail shark *C. porosus*. Interestingly, specimens of the blacktip shark *C. limbatus*, the Australian blacktip shark *C. tilstoni*, and the Queensland or graceful shark *C. amblyrhynchoides* clustered together into a single, fully supported monophyletic clade and specimens did not segregate according to species within this clade. Similarly, specimens of the silvertip shark *C. albimarginatus* and the bull shark *C. leucas* clustered together into a single but moderately supported (bv = 62) clade. The aforementioned suggests either errors in the identification of sharks prior to sequencing, ancient introgression, or recent interbreeding among specimens/species in each clade.

In contrast to that observed in the mitophylogenomic analysis, nodes towards the root of the tree were poorly supported. This is expected because short fragments of the Control Region should not have any phylogenetic information to resolve deep phylogenetic relationships in *Carcharhinus* and related genera.

In summary, although not entirely accurate (= ‘imperfect’), mitochondrial genomes assembled using long reads reliably identified the studied (and sequenced) individual of *C. falciformis* and differentiated the same individual from other closely and distantly related congeneric sharks.

## Discussion

We have successfully assembled a complete and high quality (gold-standard) mitochondrial genome for *C. falciformis* using Illumina short reads and a specialized bioinformatics pipeline exclusively developed for the retrieval of short chromosomes, including entire mitochondrial genomes (see [20]). Importantly, we used the same dataset from Galván-Tirado et al. [34] to explore if an odd ~ 939 bp long insertion observed in the original assembly [34] was a by-product of using a non-specialized assembly pipeline. Our assembled mitochondrial genome was 16,705 bp long, 934 bp shorter than the previously assembled genome by Galvan-Tirado et al. [34]. The gene order observed in *C. falciformis* was identical to other reports in the genus *Carcharhinus* [34] and the length of our assembly was most similar to that of other congeneric sharks whose mitochondrial genomes are often ~ 16,700 bp in length ([18, 35], and references therein). We concluded that the unusually long mitochondrial genome of Galván-Tirado et al. [34] was an artifact due to the use of non-specific assembly tools. We suggest that future studies should assemble mitochondrial genomes only using specialized assembly pipelines ([39] and references therein). Overall, the results from this study tell us that specialized assemblers like GetOrganelle (among a few others) should be preferred over non-specialized tools when the goal is to assemble mitochondrial genomes. Furthermore, we argue that mitochondrial genomes assembled with non-specialized bioinformatics workflows that exhibit peculiarities (i.e., unusual repeats, missing genes, and duplicated regions) need to be revisited.

The gold standard mitochondrial genome generated using short-reads Illumina sequencing data permitted us to benchmark the accuracy of the *reference-based* and de novo assembled mitochondrial genomes in *C. falciformis* using nanopore long reads exclusively. We used the same dataset of Johri et al. [35, 36] but employed different de novo and *reference-based* bioinformatics pipelines specifically developed for the rapid retrieval of mitochondrial genomes using long reads exclusively [20]. All of the workflows used in this study assembled a circular mitochondrial chromosome, as indicated after comparison of each of these long-reads assemblies with the ‘reference’ assembly generated with Illumina short reads. Furthermore, the accuracy of each long-reads assembled mitochondrial genome was high. All workflows circularized the genome with relatively high coverages (20–52.52x) and sequence (mitochondrial genome) identity, as measured by p-distance, was very high. Differences in accuracy among mitochondrial genomes assembled with the different pipelines used in this study, either de novo or *reference-based*, were minimal. These results agree with previous studies examining the accuracy of assembled chromosomes from long-reads nanopore sequences exclusively. For instance, Baeza [20] detected a slight decrease in accuracy when the mitochondrial genome of the spiny lobster *P. argus* was assembled using the program Rebaler and a distantly related congeneric species. Similarly, a second recently published study that successfully assembled chloroplast genomes using (nanopore) long reads reported decreasing accuracy when the reference genome was from a distantly related species [48]. Altogether, the information above confirms that nanopore sequencing data exclusively can be used to assemble complete and highly accurate (but not perfect, see below) mitochondrial genomes using both de novo and *reference-based* pipelines.

Benchmarking of assemblies using long reads with reference genomes obtained with Sanger or Illumina short reads is rarely reported (see [20, 48, 49]). In this study, we provided a quantitative comparison of error types in mitochondrial genomes assembled using long reads. By far, the errors that were most often observed were insertions in homopolymer runs, in agreement with that observed by the few other studies that have reported a detailed analysis of error type for small chromosomes assembled relatively short genomes [i.e., in chloroplast genomes – [48, 50], in mitochondrial genomes – [20], in bacterial genomes – [49]]. This type of error (insertions in homopolymer runs) is common in nanopore sequencing [49, 50]. We expect that this detailed report on error type will assist with the optimization of bioinformatics workflows for the de novo assembly of mitochondrial genomes using 3rd generation sequencing technologies exclusively. Importantly, the initial error rate of long-read nanopore sequencing has steadily diminished during the last few years [51]. As the authors write this paper, ONT is testing new chemistry that can produce Q20+ single pass raw read accuracy. The base-caller software Guppy is also expected to continue improving in the coming years (www.nanoporetech.com). Further development of nanopore sequencing technology is likely to result in the assembly of complete and totally accurate chromosomes (mitochondrial, plastic, nuclear) in the near future.

### Mitophylogenomics and barcoding studies using long-reads assembled mitogenomes

We assembled complete and highly accurate mitochondrial genomes in the silky shark *C. falciformis* using long-reads nanopore sequencing exclusively with the addition of different bioinformatics workflows. Although highly accurate, the assemblies were not perfect. Annotation of the different long-reads assembled mitochondrial genomes with the pipelines MITOS2 and MitoFish demonstrated that the few observed errors resulted in stop codons that disrupted the ORF of nearly every PCG, in line with that observed in [20]. In some cases, the errors resulted in the annotation pipelines missing short PCGs (i.e., *atp8*). Importantly, the observed sequence errors might constrain the reliable identification of a sequenced specimen such as *C. falciformis* and might also inhibit the differentiation of the same specimen from others belonging to the same genus in a phylogenomic and/or barcoding analysis. On the other hand, even if not fully accurate, the assembled mitochondrion or particular genes might be useful for mitophylogenomics and barcoding studies. If that was the case, and in line with [20], we predicted that entire mitochondrial genomes or fragments of them (i.e., D-Loop/Control Region) assembled with de novo and *reference-based* pipelines will cluster together with the ‘gold-standard’ short-reads assembled mitochondrial chromosome as well as with other entire or partial (CR) mitochondrial genomes from the same species available in GenBank. Furthermore, the complete or partial mitochondrial genomes will be expected to segregate from others belonging to closely related species in the same genus *Carcharhinus*. Supporting the view that long-read assembled mitochondrial genomes are suitable for mitophylogenomics and barcoding research, in a first mitophylogenomic analysis that used all PCGs, the short-reads assembled reference genome and all of the long-reads assembled mitochondrial genomes clustered together into a single, fully supported monophyletic clade. Interestingly, the analysis positioned *C. falciformis* (reference [short-reads] plus all long-reads assemblies) as sister to the blue shark *Prionace glauca*, in line with that reported by a recent study that used complete mitochondrial genomes with a smaller number of shark species ([18, 35] and references therein). Additionally, in our second barcoding analysis that used the D-Loop/Control Region, the short-reads assembled reference genome and the totality of the long-reads assembled mitochondrial genomes with 15 other sequences belonging to *C. falciformis* available in Genbank clustered together into a fully supported monophyletic clade. The *C. falciformis* monophyletic clade segregated from other clades, comprised of 457 sequences belonging to other closely related (congeneric) species. Altogether, the information above allows us to conclude that long-reads assembled mitochondrial genomes, although imperfect, are consistently able to identify the sequenced individual as belonging to *C. falsiformis* and genetically differentiate it from other closely related species.

Together with other recent studies [20, 48, 49], our results suggest that nanopore long reads (with further development) and customized pipelines can be used to address major conservation and management issues in marine organisms, including the silky shark *C. falciformis*, and likely, other closely and distantly related species experiencing similar conservation problems. For instance, nanopore technology (after further improvement of initial read error) and ancillary bioinformatics pipelines can be used to quickly test in situ mislabeling in the shark fin fishing industry and thus, improve surveillance protocols, law enforcement, and the regulation of this fishery [52]. Overall, in situ mislabeling in the shark fin fishing industry is expected to inform and improve conservation strategies in silky sharks and other species experiencing major conservation issues [52].

Second, we argue that nanopore long read sequencing technology has the potential to democratize genomic research in middle- and low-income countries by breaking cost-barriers; it can provide relatively cheap and quick access to high-throughput sequencing technologies to researchers in those countries. Importantly, library preparation is relatively rapid and straightforward for nanopore sequencing, and the sequencing device itself is inexpensive compared to PacBio, the only other current long-read sequencing technology alternative to nanopore [17, 53]. Nanopore sequencing can be used for the rapid retrieval of genomic information (i.e., mitochondrial genomes) in studies surveilling the shark-fin fishing industry and studies exploring population genomic structure and connectivity among close and distant populations in silky sharks. The understanding of connectivity, demographic history, source-and-sink metapopulations dynamics, and genomic diversity, among others, is expected to inform and improve the implementation and design of marine protected areas for *C. falciformis* and other sharks ([18, 52] and references therein). As stated above, these studies need to be implemented in situ and in collaboration with local scientists to avoid ‘parachute science’, a pervasive practice in the USA and Europe [54]. Long-reads nanopore technology can be used to deliver rapid and cheap genetic marker retrieval to international teams of scientists interested in contributing to environmental problem solutions using inclusive research strategies [54].

This study differs in several ways from that of Baeza [20], the only previous study that has benchmarked long-reads assembled mitochondrial genomes generated from ONT long-read sequences with a short-reads mitochondrial genome assembly from Illumina short-reads [20]. First, in this study we have used the software Guppy v. 3.3.1 (instead of Albacore used in [20]) to improve base-calling accuracy. The use of Guppy with updated versions of the different programs comprising the pipeline used in this study (compared to the versions used in [20]) was expected to increase base-calling accuracy, as well as the accuracy of the final long-reads mitochondrial genome assemblies. Importantly, we observed a greater number of total mitochondrial genome assembly errors in this study (mean [S.D.] = 85.78 ± 15.87, range = 65–110) compared to that in [20] (mean [S.D.] = 60.5 ± 9.34, range = 51–77). In other words, even though we have used a more sophisticated or advanced pipeline, the final mitochondrial genome assemblies accuracy did not improve but rather decreased. Furthermore, we observed that the most common errors in this study were insertions (e.g., mostly 1 pb insertions) at the flanks of homopolymer regions, while in [20], the most common error were due to deletions at the flanks of homopolymer regions. We do not know which conditions explain the observed differences. Nonetheless, the observed dissimilarities between this study and that of Baeza [20] argue in favor of conducting additional research to understand the parameters and conditions driving the accuracy of assemblies (as well as type of errors) when relying solely on ONT long read sequencing. During the preparation of this manuscript, we are aware that ONT has started to introduce new cartridges and chemistries that are expected to increase the accuracy of long read sequencing. Furthermore, the software Guppy is constantly improving in terms of base-calling accuracy (Oxford Nanopore Technologies). We think that in the near future (5–10 years), ONT long-read sequencing alone will be fully reliable for the assembling of fully accurate relatively short (mitochondrial, viral), intermediate (bacteria), and long genomes (nuclear genomes) in a wide variety of organisms.

## Conclusion

Using nanopore long-read sequencing technology and specialized bioinformatics pipelines, we have assembled a complete and highly accurate mitochondrial genome belonging to the silky shark *C. falciformis*. The silky shark is an ecologically relevant species in pelagic environments and is heavily targeted by a profitable fishery worldwide. The long-reads assembled mitochondrial genomes were highly accurate, reliably identified the sequenced individual as belonging to *C. falciformis,* and differentiated the same individual from others belonging to congeneric species. This study will facilitate the transferring of high throughput genomic technologies to middle- and low-income countries worldwide, allowing collaboration and cooperation among international teams of researchers interested in conducting inclusive research on the conservation biology of vulnerable and endangered sharks.

## Methods

### Sampling of the silky shark *Carcharhinus falciformis*

One adult individual (already euthanized by fishermen) was bought from fishermen near Playa Palo de Santa Rita Sur (24.1414° N, 110.3417° W), La Paz, Baja California Sur, Mexico. The specimen was transported to the Laboratorio de Organismos Acuaticos, Instituto de Ciencias del Mar y Limnologia (ICMyL), Universidad Autonoma de Mexico (UNAM). Muscle was extracted from the specimen with forceps, and the tissue was immediately preserved in 95% ethyl alcohol. Total genomic DNA (gDNA) was extracted from muscle tissue using an EZNA Genomic DNA Purification kit (Omega Bio-Tek, Norcross, GA). The gDNA sample was then transported to the Georgia Genomics and Bioinformatics Core, University of Georgia, Athens, GA, USA, where library preparation and Illumina paired-end shotgun sequencing were carried out.

### Illumina short reads library preparation and sequencing

An Illumina® library was prepared by shearing ~ 1 μg of gDNA (using a Covaris instrument) following the standard protocol of the Illumina Truseq DNA Library Preparation kit using a multiplex identifier adaptor index (Illumina). Illumina sequencing was conducted on a MiSeq v2® platform using a 2 × 500 cycle to produce 250 pb paired-end reads. A total of 478,450 PE reads was generated by the sequencing facility (available in the short-read archive [SRA] repository [accession number SRR18001997]) at GenBank. These reads were used for the mitochondrial genome assembly of *C. falciformis*.

### Short-reads mitochondrial genome assembly of the silky shark *Carcharhinus falciformis*

The mitogenome of *C. falciformis* was de novo assembled using the pipeline GetOrganelle v1.6.4 [39]. A fragment of the *cox1* PCG available in GeneBank (MK308176) was used as a reference. A relatively large word (kmer) size of 39 was used during the assembly. Next, the web servers MITOS2 (http://mitos2.bioinf.uni-leipzig.de/index.py) [40] and MitoFish v3.63 (http://mitofish.aori.u-tokyo.ac.jp/) [41] were used to annotate the newly assembled mitochondrial genome with the vertebrate genetic code. Manual curation of the in silico annotated mitochondrial genome, including start and stop codon corrections, were conducted using the Expasy translate tool (https://web.expasy.org/). Visualization of the mitochondrial genome was conducted with MitoFish v3.63 [41]. This short-read assembled mitochondrial genome represents the ‘ground truth’ or ‘golden standard’ reference (i.e., the trusted reference) which we used for benchmarking the quality (i.e., accuracy) of the *reference-based* and de novo assembled genomes using nanopore long reads, exclusively.

### Silky shark *Carcharhinus falciformis* nanopore long reads dataset

We assembled the mitochondrial genome of *C. falciformis* using long reads exclusively and benchmarked the accuracy of the long-reads assembled genomes by comparing them to a ‘gold’ standard mitochondrial genome generated using short-read Illumina sequencing data (see above) as in [4]. We used the same dataset of Johri et al. [18, 35] but employed different de novo and a *reference-based* bioinformatics pipelines specifically developed for the rapid retrieval of mitochondrial genomes using long reads exclusively (see [20] for an example). Details on specimen collection, gDNA extraction, library preparation, sequencing in a MinION ONT device, and raw signal (FAST5 files) base-calling with the software Guppy v. 3.3.1 (Oxford Nanopore Technologies) can be found in [18, 35]. A total of 74,536 nanopore long reads were downloaded from GenBank (SRA accession number SRX4977038) and used for assembling the mitochondrial genome of *C. falciformis* using different de novo and a *reference-based* bioinformatics pipelines.

### Quality control of long reads

First, we used the software Porechop (https://github.com/rrwick/Porechop) to trim adapters from the ends of the reads and to split sequences with internal adapters into two. Next, we used the program fastp [55] to quality-filter the reads and retain only those sequences with Q-score ≥ 6. The aforementioned QC step resulted in a total of 50,780 ‘clean’ reads that were used for the de novo and *reference-based* assembly of the mitochondrial genome of *C. falciformis*.

### De novo long-reads mitochondrial genome assembly of the silky shark *Carcharhinus falciformis*

**Fig. 6:**
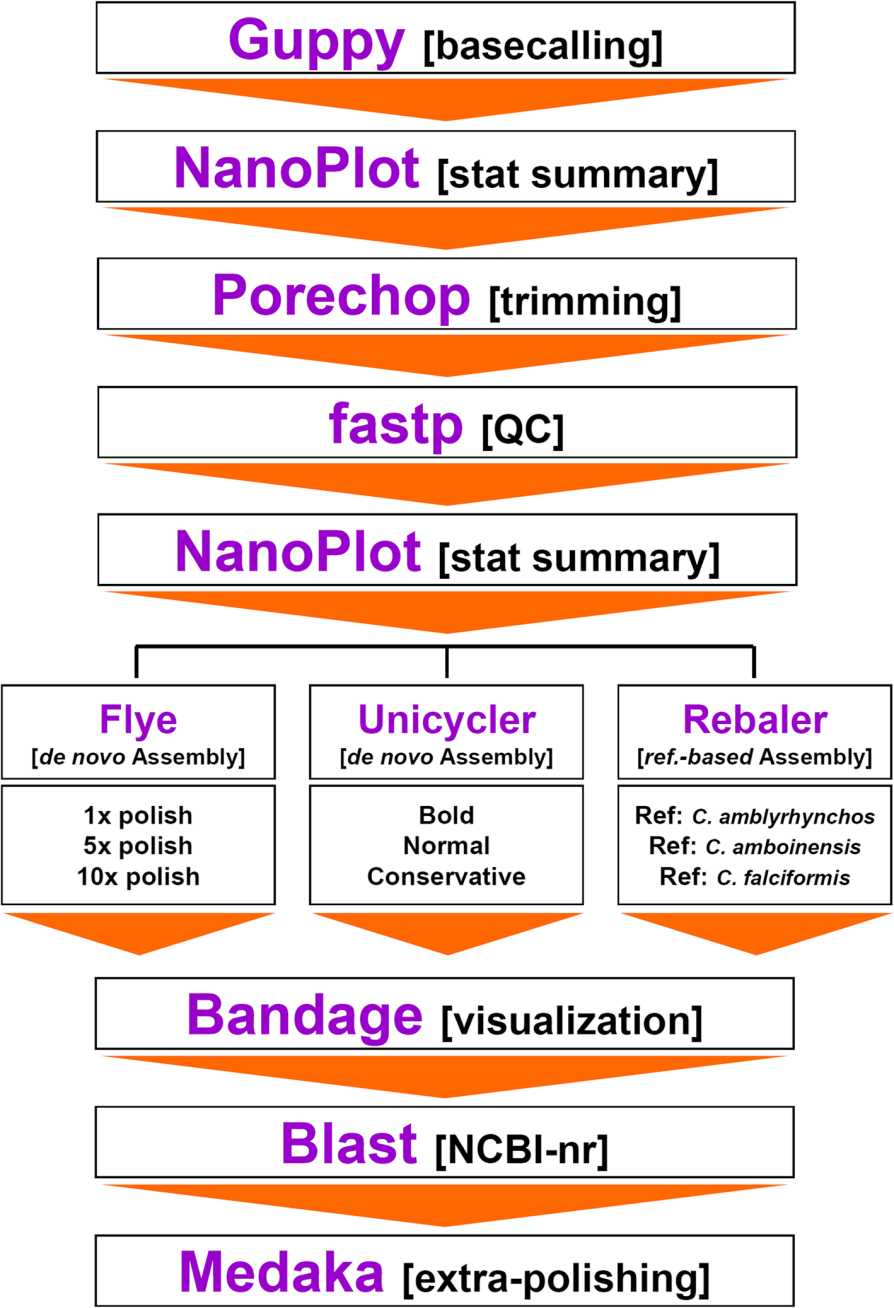
Bioinformatics pipeline to assemble the mitochondrial chromosome of the silky shark *Carcharhinus falciformis* using nanopore long reads exclusively

We de novo assembled the mitochondrial chromosome of *C. faciformis* using the pipelines Unicycler 0.4.8–1 [42] and Flye 2.8–0 [43] (Fig. 6).

We ran Unicycler as in [20] using three different modes: normal (the default), bold, and conservative. The bold mode is the most likely to assemble complete genomes but conveys the greatest risk of misassembly, while the conservative mode has a very low risk of misassembly but it is least likely to produce a complete assembly. Finally, the normal mode is intermediate with respect to completeness and misassembly risk (see https://github.com/rrwick/Unicycler).

Flye polishes a final set of assembled contigs with the program Flye polisher [42]. By default, a single polishing iteration is run by Flye. In this study, we ran Flye with 1, 5, and 10 iterations with the goal of improving the final assembly. We assumed that with increased iterations, a larger number of errors would be corrected by Flye polish.

After the assembly step, either with Flye or Unicycler, we used the program Bandage [46] to visualize the assembly graph produced by the two de novo pipelines as in [4]. If Flye and/or Unicycler successfully assembled and circularized the mitochondrial chromosome of *C. falciformis*, we predicted that a circular sequence ~ 16 kpb long would be observed among the contigs in the assembly graph. We blasted any observed circular assembled contigs from each assembly pipeline to the nucleotide non-redundant database in NCBI’s GenBank and calculated the statistical significance of the matches.

Lastly, we used the software Medaka v 1.0.3 (https://github. com/nanoporetech/medaka) [45] to apply a final ‘extra polishing’ employing the model r941_prom_high_g330 to the different mitochondrial genomes assembled with the two de novo pipelines above as in [4, 20]. Medaka uses neural networks to a pileup of individual nanopore reads against a draft assembly to create a new final consensus sequence. In this study, the draft assembly corresponds to the final assembly produced by either Flye or Unicycler while the new final consensus sequence corresponds to the extra-polished mitochondrial genome assembly.

### *Reference-based* long-reads mitochondrial genome assembly of *Carcharhinus falciformis*

*Reference-based* assemblies of the *C. falciformis* mitochondrial genome were conducted using the pipeline Rebaler (https://github.com/rrwick/Rebaler) [44] (Fig. 6). We executed Rebaler using three different reference genomes: *C. amboinensis* (NC_026696), *C. amblyrhyncos* (MT663280) and the short-read mitochondrial genome of *C. falciformis* assembled during this study. *Carcharhynus amboinensis* is less distantly related to *C. falciformis* that *C. amblyrhyncos* [56]. The choice of different mitochondrial genomes above allowed us to check for differences in accuracy of the final assembly due to genetic differences among reference genomes. Also, we ran Rebaler with the option ‘circular = true’ indicating that the reference genome was circular so that Rebaler ‘rotated’ contigs between polishing rounds to ensure improved accuracy of the final assembled mitochondrial genome (https://github.com/ rrwick/Rebaler). A final ‘extra-polishing’ step with the software Medaka was applied to each mitochondrial genome assembled with Rebaler (https://github.com/nanoporetech/ medaka)

### Evaluation of accuracy in long-reads mitogenome assemblies

We evaluated the quality (i.e., accuracy) of each long-read assembled mitochondrial genome (without and with ‘extra polishing’ using Medaka) using four metrics as in [4, 20]: number of contigs, assembly length, coverage, and identity. We implemented p-distance (patristic distance, uncorrected) as a measure of sequence identity. Low and high *p*-values indicate, respectively, low and high sequence accuracy. Identical short-reads reference and long-reads assembled mitogenomes are indicated by a p-distance value equal to zero. To calculate Patristic distance (p-distance) between each long-reads assembled mitochondrial genome and the short-reads assembled reference genome, we aligned mitochondrial genomes assembled using long reads to the short-reads reference genome with the program Muscle [57] as implemented in MEGAX [58].

Lastly, long-reads assembly error was quantified in a manner similar to that of [4, 20]. After each long-reads assembly (without and with ‘extra polishing’ using the program Medaka) was aligned to the reference assembly, errors were classified as single, double, triple, quadruple, quintuple, sextuple, or septuple “homopolymer insertions’ or ‘homopolymer deletions’ if the error added or removed, respectively, a single, two, three, four, five, six, or seven bases from a homopolymer (i.e. multiple consecutive appearances of the same nucleotide) regions two or more bases in length [4]. Other errors that did not fit with any of the categories mentioned above were classified as ‘simple substitution’, ‘single insertion’, ‘short insertion (<5 bp)’, ‘single deletion’, and ‘short deletion (<5 pb)’. We note that the gold standard short-reads mitochondrial genome assembly used in this study was retrieved from a specimen different than that from which ONT long-reads were obtained. Taking into account that (i) the rate of molecular evolution (nucleotide substitution) in mitochondrial genomes belonging to elasmobranchs is low ([36] and references therein), and (ii) deletion and/or insertions at the flanks of homopolymer regions represent the overwhelming majority of errors previously detected in contigs assembled with ONT long-read [20], we expect to observe an obvious error-signal when the different long-reads assemblies and the short-reads gold standard assembly used in this study are compared, even if they are retrieved from different conspecific individuals.

### Annotation of mitochondrial genomes assembled using long reads

We annotated each *reference-based* and de novo long-reads assembled mitogenome with the online pipelines MITOS2 [40] and MitoFish [41] using the vertebrate mitochondrial code. The presence/absence of stop codons causing truncated PCGs (i.e., with interruptions in the open reading frame) was recorded. The latter constitutes an additional proxy for long-read assembly accuracy estimated in this study.

### Phylogenomic and barcoding utility of long-reads mitochondrial assemblies

We determined the utility of the long-reads, newly assembled mitochondrial genomes for phylogenomics and barcoding research. Following [4, 20], we predicted that, in both mitophylogenomic and barcoding analyses, the mitochondrial genomes assembled using long reads will cluster with the reference short-reads assembly genome and will segregate from other mitochondrial genome sequences belonging to closely and distantly related species (i.e., in the same genus and family) available in Genbank.

First, to test the phylogenomic utility of long-reads mitochondrial assemblies, the mitochondrial genomes (*N* = 16) belonging to different species in the genus *Carcharhinus* were retrieved from GenBank (available as of 05 252,021). *Carcharhinus* has diversified since the middle Eocene, about 45 Myr ago [59, 60]. We also retrieved mitochondrial genomes from the genus *Glyphis* (*n* = 5 species), *Lamiopsis* (*n* = 2), *Sphyrna* (*n* = 4), and *Loxodon* (*n* = 1) that were used as outgroups in the analysis. Lastly, we retrieved the mitochondrial genomes of the whitetip reef shark *Triaenodon obesus* (NC026287) and the blue shark *Prionace glauca* (NC022819) considering that previous studies clustered these two species within the genus *Carcharhinus* [56]. Next, all 13 PCGs plus the two ribosomal RNA genes (12S and 16S) from each long-read assembled mitochondrial genome of *C. falciformis* and the short-read assembled reference genome of *C. falciformis* plus the 29 mitogenomes retrieved from Genbank were aligned using the software Muscle (with default options) as implemented in the program MEGA X. The final alignment, comprised of 14,245 bp, was provided to the web server IQ-TREE 1.6.12 (http://iqtree.cibiv.univie.ac.at/) for Maximum Likelihood (ML) analysis [61]. The software ModelFinder [62], as implemented in IQ-TREE, was used for selecting a base substitution model that best fits each dataset. The optimal models found by ModelFinder (selected with the Bayesian Information Criterion) were the TN + F + G4 for *atp6*, HKY + F + G4 for *atp8*, TIM2 + F + R3 for *cox1,* TN + F + I + G4 for *cox2,* TIM2 + F + I + G4 for *cytb, nad1, nad2, nad4, nad5,* 12S rRNA DNA, and 16S rRNA DNA, TIM2 + F + R3 for *cox3*, TIM2 + F + G4 for *nad3,* TN + F + I + G4 for *nad4l*, and TIM + F + G4 for *nad6*. A total of 1000 bootstrap replications were conducted to estimate support for each node in the Maximum Likelihood tree [61].

Second, to test the barcoding usefulness of long-reads mitochondrial assemblies, a total of 457 D-Loop/CR sequences belonging to the genus *Carcharhinus* plus 19 other sequences used as outgroup (*Galeocerdo* spp. = 5 sequences, *Glyphis* spp. = 5, *Lamiopsis* spp. = 2, *Loxodon macrorhinus* = 1, *Prionace glauca* = 1, *Sphyrna* spp. = 4, *Triaenodon obesus* = 1) were retrieved from GenBank (available as of 05 152,021). Next, the software Clustal Omega [63] as implemented in the web server EMBO (https://www.ebi.ac.uk/Tools/ msa/clustalo/) was used to align all of the retrieved sequences plus the CR fragment from all of the long-read and short-read (reference genome) assemblies using the default parameters. The final alignment consisted of 1188 bp. Next, the aligned dataset was exported to the web server IQ-TREE 1.6.12 (http://iqtree.cibiv.univie.ac.at/) for Maximum Likelihood (ML) analysis [61]. Selection of a base substitution model that best fits each dataset was conducted with ModelFinder [62] as implemented in IQ-TREE. The optimal models found by ModelFinder (selected with the Bayesian Information Criterion) was the TIM + F + R4. All the parameters used for the ML analyses were those of the default options in IQ-TREE and 1000 bootstrap replications were conducted to estimate support for each node in each Maximum Likelihood tree [61].

We note that the great majority of the complete mitochondrial genomes and CR fragments deposited in GenBank are assembled using Sanger sequencing or Illumina short-reads, (https://www.ncbi.nlm.nih.gov/genbank/).

## Data Availability

All datasets on which the conclusions of the manuscript rely are presented in the main paper. Short read sequences are available in the short-read archive (SRA) repository (Bioproject ID: PRJNA772885; Biosample accession: SAMN25885101; SRA accession: SRR18001997) at GenBank. Long read sequences are available in the SRA repository (accession number SRX4977038). Mitochondrial genome assemblies generated in this study are available in the short-read archive (SRA) repository (SRA accessions: OM885432 - OM885450). The specimen is deposited at the ‘Laboratorio de Genética de Organismos Acuáticos’. Instituto de Ciencias del Mar y Limnología, Universidad Nacional Autonoma de Mexico, Mexico (Curator: Dr. Píndaro Díaz Jaimes).

## Abbreviations

DNA: Deoxyribonucleic Acid
ONT: Oxford Nanopore Technologies
gDNA: Genomic DNA
PCG: Protein-coding genes
PCR: Polymerase chain reaction
RNA: Ribonucleic Acid
tRNA: Transfer RNA
rrnS: 12S ribosomal RNA
rrnL: 16S ribosomal RNA
WGS: Whole genome sequencing
